# Computationally Efficient Algorithm for Modeling Grain Growth Using Hillert’s Mean-Field Approach

**DOI:** 10.3390/ma17102341

**Published:** 2024-05-15

**Authors:** Shabnam Fadaei Chatroudi, Robert Cicoria, Hatem S. Zurob

**Affiliations:** Department of Materials Science and Engineering, McMaster University, Hamilton, ON L8S 4L8, Canada; fadaeics@mcmaster.ca (S.F.C.); cicorir@mcmaster.ca (R.C.)

**Keywords:** modeling, grain growth, mean-field modeling, micro-alloyed steels, grain-size distribution, upsampling algorithm

## Abstract

To investigate the interconnected effects of manufacturing processes on microstructure evolution during hot-rolling, a through process model is required. A novel numerical implementation of the mean-field approach was introduced to efficiently describe the grain growth of larger systems and extended durations. In this approach, each grain is embedded within an average medium and interacts with the average medium, thus avoiding the complexities of individual grain interactions. The proposed upsampling approach dynamically adjusts the simulation grain ensemble, ensuring efficiency and accuracy regardless of the initial number of grains present. This adaptation prevents undersampling artifacts during grain growth. The accuracy of the model is verified against analytical solutions and experimental data, demonstrating high agreement. Moreover, the effects of different initial conditions are successfully investigated, demonstrating the model’s versatility. Due to its simplicity and efficiency, the model can be seamlessly integrated into other microstructure evolution models.

## 1. Introduction

The production of modern line pipe steels involves various thermomechanical processing steps that impact microstructure evolution during the hot-rolling process. There is great demand within the steel industry to develop physics-based through-process models for microstructure evolution during thermomechanical processing, including the interactions between processes [1,2,3,4,5,6]. The simplicity of the model is crucial, as it enables computationally efficient numerical schemes. For instance, the crystal growth model introduced by Elder et al. [7] achieved simulations that were many orders of magnitude faster than other atomistic methods. These models have the potential to accelerate the development of new products as well as improve existing products and processes, provided that a large number of simulations can be carried out efficiently to explore the effects of chemistry and process parameter changes. Understanding detailed microstructure evolution aspects such as grain size, dislocation density, precipitate radius, volume fraction, and solute content is necessary to determine flow stress during rolling [8,9,10]. Moreover, this information can be used to predict room temperature properties, including yield stress, ductile–brittle transition temperature (DBTT), and grain-size distribution [9,11]. One of the key processes to capture during the hot-rolling of steels is austenite grain-size evolution during reheating and hot-rolling.

During grain growth, a polycrystalline microstructure can undergo changes with respect to grain size, grain shape, orientation, and size distribution. A quantitative description of this process is essential for optimizing a wide range of processes in which the material spends time at high temperatures. The driving force for grain growth arises from the grain boundary curvature of adjacent grains of dissimilar sizes. In this situation, larger grains will grow at the expense of small ones—a process known as grain growth. Grain growth can occur in different regimes, referred to as “normal” and “abnormal” growth. Normal grain growth is typically characterized by parabolic growth kinetics and a self-similar grain-size distribution [12]. On the other hand, in abnormal grain growth (often referred to as secondary recrystallization), a small number of larger grains emerge, gradually consuming a matrix of smaller grains. A bimodal grain-size distribution is observed during abnormal grain growth [13,14,15].

Various analytical models exist for normal grain growth. In the early 1950s, Burke and Turnbull [16] proposed a treatment that predicted parabolic grain-growth kinetics. Their treatment modeled boundary migration as a process involving atomic jumps across the boundary, driven by pressure differences which arise from surface curvature. Soon after, C.S. Smith [17] highlighted the role of surface tension equilibria and topological requirements during grain growth. He proposed that a tendency exists towards a consistent grain-size distribution (GSD), which was shown experimentally to be a log-normal distribution. Hillert adapted the treatments of particle coalescence [5,6,7,8] to grain growth and was able to arrive at a self-similar grain-size distribution for normal grain growth.

The grain-growth treatment by Hillert [1] as well as those developed by Feltham [18] and Louat [19] can be described as mean-field models (MFM). The mean-field approach addresses the evolution of the size of an individual grain situated within an environment/medium whose properties are obtained by averaging the properties of the entire array of grains. Observing microstructure evolution in materials through experimental studies on grain growth can be time-intensive and costly. Therefore, a quantitative understanding of microstructure evolution during this stage is essential. Numerical simulation provides a practical alternative to address this challenge. In recent years, numerical implementations of the mean-field treatment of grain growth have been introduced. The advantage of the numerical approach is that it can handle grain-size evolution over a wide range of conditions, including situations when the grain-size distribution is not the steady-state distribution. Some of the recent work in this area includes that of Enomoto et al. [20], who employed an MFM based on the Abbruzzese and Lucke model [21,22]. The simulated grain-size distribution was compared to the measured distributions for relatively short times of up to 100 s. Wu et al. developed MFM for normal grain-growth simulations, successfully validated against parabolic growth theory, and obtained a steady-state Hillert distribution. However, the outputs did not align well with the experimental data [23]. Maire et al. [24] evaluated the MFMs of grain growth against 3D full-field simulations. Their study demonstrated the adaptability of the Hillert model, particularly due to its incorporation of the initial grain-size distribution (GSD) and a discrete representation of the microstructure. A recent review by Roth et al. [25] compared experimental grain-growth data to the predictions of numerical MFM simulations based on the approaches of Hillert [26], Abbruzzese et al. [27], and Maire et al. [28]. It was found that the Hillert model generated more accurate predictions of the GSD, with a simpler average medium description. Another study [29] demonstrated the potential to couple MFM grain-growth simulations and Thermo-Calc in order to capture particle-pinning effects.

In contrast to mean-field models, full-field models aim to provide a comprehensive description of the microstructure by capturing the complete topology at the polycrystal scale. Atomistic simulations demonstrate anisotropies in boundary energy and mobility. However, simulations of microstructural evolution suggest that anisotropy in boundary mobility has a minimal impact on a microstructure’s stochastic evolution, possibly affecting only the rate of evolution. Conversely, anisotropy in grain boundary energy significantly alters the topology of the polycrystalline microstructure [30,31]. A topological investigation of 3D growth by tracking the number of faces of grains discovered deviations in grain-size distribution exhibiting relatively longer tails of large grains [32]. Several full-field methodologies have been effectively employed to simulate the process of grain growth, such as the Monte Carlo Potts model [33,34,35,36,37], the Surface Evolver [38,39,40], the Vertex Models [41,42,43,44], the Front-Tracking Models [45,46,47], and the Phase-Field Models [32,48,49,50,51]. When the main goal of the simulation is to track the grain-growth kinetics and grain-size distribution for equiaxed grains, the MFM can provide an adequate description of the microstructure with competitive computation times compared to full-field models [52,53,54,55].

In this study, we have opted to employ a mean-field model to study 3D grain growth assuming isotropic grain boundary energy and mobility, a constant temperature, and the absence of precipitation. Unlike other numerical approaches, the MFM allows for a simplified representation of the complex interactions occurring between individual grains, allowing for the simulation of larger systems over extended time periods [52]. The benefit of the mean field’s description lies in its simplicity; however, it cannot capture spatially heterogeneous microstructural effects like segregation and the topology of the polycrystalline microstructure [56,57]. In order to accurately simulate grain growth, it is essential to maintain a substantial number of grains during the simulation [51]. If one considers a constant simulation volume, the number of grains within this volume will decrease as grain growth progresses, and, eventually, the number of grains present will not be sufficient to allow the accurate calculation of the grain-growth kinetics and grain-size distribution. Conversely, the opposite situation can occur in the context of recrystallization. Repeated cycles of recrystallization could lead to the introduction of an enormous number of new grains within the simulation volume, leading to a system which is too large to efficiently simulate, which will be investigated in our next paper. In this work, we introduce a numerical algorithm that dynamically adapts the size of the simulation volume in order to maintain both the accuracy and efficiency of the grain-growth simulation.

## 2. Modeling Framework

**Figure 1:**
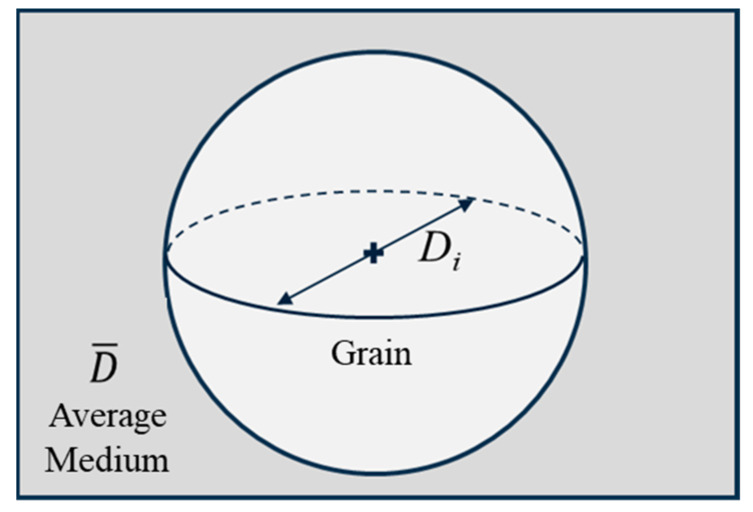
Illustration of a grain embedded in an average medium.

The polycrystal material is represented by a population of grains evolving within the average medium, with properties representative of the aggregate referred to as the mean field [58]. This approach closely resembles the self-consistent models frequently employed in the field of continuum mechanics for inhomogeneous materials. In this model, a set of spherical grains is used to represent the material, and each individual grain is described using a set of variables at any given moment. In our case, the variable of interest is size (*D_i_*), as shown in Figure 1. Each grain is surrounded by a uniform matrix, with properties obtained by averaging those of all the grains ($\bar{D}$). In the present implementation, computational efficiency is maintained by binning grains with a similar size. A grain class or bin is a set of grains with identical characteristics. These bins or classes have a size and a frequency associated with them. Each class enables the definition of a representative grain, possessing the characteristics associated with its corresponding grain class.

The MFM does not keep track of the neighbours of an individual grain. Instead, each grain is compared to the average medium. Thus, the average growth rate for a specific class is determined by the difference in the radii of curvature between the current class and the average medium, as follows [26]:

(1) $$\frac{dR_{i}}{dt}=\alpha M\sigma (\frac{1}{R_{Cr}}-\frac{1}{R})$$

where *M* is the grain boundary mobility, *σ* is the grain boundary energy, *R_Cr_* is the critical radius (*R_cr_ =* $\bar{R}$ in 2D, and *R_cr_ =* 9/8 $\bar{R}$ for a 3D system where $\bar{R}$ is the mean radius), and *α* is a geometrical constant (*α* = 0.5 for a 2D, and *α* = 1 for a 3D system) [26]. The grain structure can be represented by a dynamic grain number, *N_i_*, describing the number of grains with radius *R_i_* at time *t*. In order to ensure volume conservation during normal grain growth ($\frac{dV}{dt}=$ 0), the following is required:

(2) $$4\pi \sum \frac{{N_{i}R}_{i}^{2}dR_{i}}{dt}=0$$

By substituting the $\frac{dR_{i}}{dt}$ into $\frac{dV}{dt}$, the critical radius is expressed as follows [59,60]:

(3) $$R_{cr}=\frac{\sum_{i}{N_{i}R}_{i}^{2}}{\sum_{i}N_{i}R_{i}}$$

The critical radius is substituted into Equation (1) to calculate the growth rate of each grain. Each individual grain bin is updated at each time step by integrating the growth rate equation using Euler’s method:

(4) $$R_{\left(i,t+dt\right)}=R_{\left(i,t\right)}+\frac{dR_{\left(i,t\right)}}{dt}∆t$$

As grain growth progresses, the total number of bins in the system will decrease. If the number of bins drops critically low, the above algorithm will lead to numerical artefacts and reliable estimates of the average grain size, and the grain-size distribution would not be obtained. To avoid this issue, an “Upsampling Algorithm” has been introduced. The concept of the upsampling algorithm has been widely employed in image processing for multiple purposes, notably for resolution enhancement and performing interpolation [61,62,63,64,65]. In our study, the basic idea of the developed algorithm is to consider that our volume sample size is the limitation that introduces these sampling artefacts. Therefore, we can speculate that other grain-size classes (bins) would exist in the gaps of ensemble if our volume sample window were to be different. By adding new bins within these gaps while conserving the simulation volume, we can mitigate the undersampling issue. The algorithm begins by ordering the grain bins by size and identifying the largest gap between adjacent bins. A new grain bin is then introduced inside the gap. This algorithm acts as a smoothing statistical function to ensure that a sufficient number of grain bins exist throughout the simulation, to ensure that Equations (1)–(4) can accurately predict the grain-growth kinetics.

In order to introduce new bins, a sampling kernel function is implemented.

**Figure 2:**
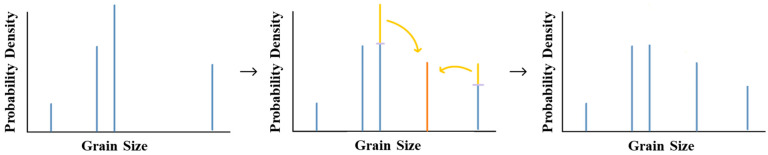
A visual demonstration of the upsampling algorithm, illustrating the insertion of additional bins during grain growth to maintain the desired bin distribution.

This function essentially acts as a window function, analyzing neighboring bins to determine the characteristics of the new bin. This function can take on a wide variety of forms, such as a Box function or a Gaussian function. However, it must adhere to the constraint of conserving volume throughout the process. For simplicity, we opt for a kernel that utilizes data from the nearest two neighbors. The volume of the newly introduced bin is determined by the sum of 1/3 of the volume of the bin immediately smaller than it and 1/3 of the volume of the bin immediately larger than it. This choice of values ensures a linear transition within the gap. The corresponding volume is removed from the adjacent bins, as illustrated in Figure 2. This process is repeated until the minimum target ensemble size is achieved. This algorithm acts as a smoothing statistical function to ensure that a sufficient number of grain bins exist throughout the simulation and a smooth distribution is maintained. Furthermore, the upsampling algorithm should protect complex distribution features, such as multimodality, by conserving volume during the upsampling process. When bins are inserted between the peaks in multimodal grain-size distributions, they will possess minimal volume fractions, reflective of the small volume fractions of the bins on the outskirts of the peaks. Additionally, a restriction can be imposed on the kernel sampling window to exclude gaps in the distribution beyond a certain size threshold from consideration.

In the next step, the essential material parameters need to be identified for developing the MFM for a specific system. In order to evaluate the grain-growth kinetics against experimental data, we need to define the mobility for a specific system of interest. For a Niobium micro-alloyed steel [66,67], it has been demonstrated that the grain boundary mobility can be described using Cahn’s solute drag model [68]:

(5) $$M^{\prime }={\left(\frac{1}{M_{p}}+\alpha_{m}C_{Nb}\right)}^{-1},$$

(6) $$with\;\alpha_{m}=\frac{\delta N_{v}{\left(k_{b}T\right)}^{2}}{E_{b}D_{x}}\;\left(\sinh\left(\frac{E_{b}}{k_{b}T}\right)-\frac{E_{b}}{k_{b}T}\right)$$

*M*′ represents the grain boundary mobility when Nb is present, *C_Nb_* is the atomic fraction of the Niobium in solution, *N_v_* represents the number of atoms per unit volume, *E_b_* denotes the binding energy of Nb to the grain boundary, *D_x_* refers to the cross-boundary diffusion coefficient of Nb, and *k_b_* represents Boltzmann’s constant. In this equation, *M_p_* is the intrinsic mobility, which refers to the mobility of the grain boundary in the pure material. Furumai et al. and Zhou et al. [66,67] identified this with the mobility of the Nb-free C-Mn steel as follows:

(7) $$M_{p\;}(T)=\frac{A}{T}\;.\exp\left(-\frac{Q}{RT}\right)$$

where *A* = 0.2605$\;[m^{4}K/s/J]$, *Q* = 173160 [J/mol], and *R* is the gas constant [J/K/mol]. The values of *M_p_* range from $4.87\times 10^{-11}[m^{4}/s/J]$ at 1100 °C to $6.06\times 10^{-10}\;[m^{4}/s/J]$ at 1400 °C.

## 3. Results and Discussion

To start, it is necessary to validate the accuracy of the present algorithm by confirming that it can reproduce the key predictions of grain-growth theory. Once this has been demonstrated, the computational efficiency of the model will be demonstrated. The simulations are carried out in comparison with the F-C-Mn-Nb system, as described in the previous section.

**Figure 3:**
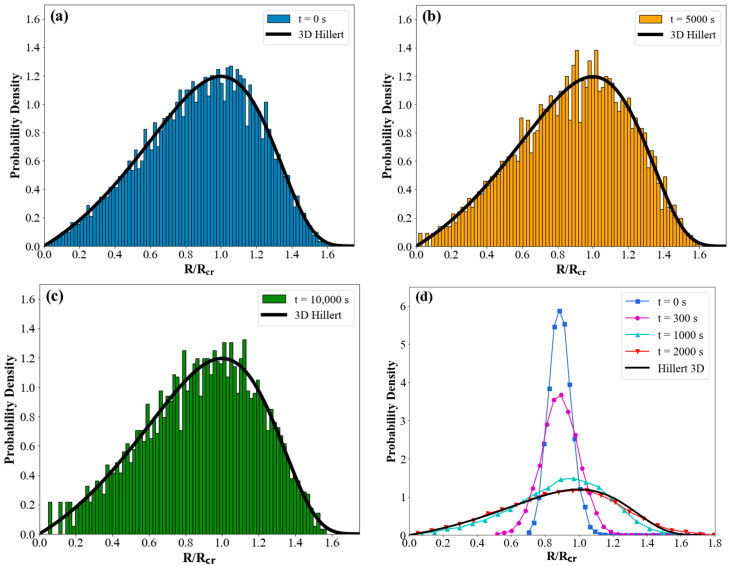
Grain-size distribution evolution of normal grain growth during 3D ideal grain growth at 1100 °C compared with theoretical Hillert GSD (black solid line) in the following conditions: (**a**) initial GSD as Hillert at t = 0 s, (**b**) t = 5000 s, and (**c**) t = 10,000 s; (**d**) initial GSD as log-normal distribution at different time steps, which, after 2000 GSD, remains unchanged, corresponding to the Hillert distribution, indicating a steady state.

Figure 3 shows the probability density of the grains (frequency of data points in each bin) versus normalized grain size (*R/R_cr_*) during grain growth to illustrate the evolution of GSD. It is important to note that all the grain-size distribution profiles represent instantaneous distributions at the specified times. For comparison, we also included the theoretical Hillert distribution function:

(8) $$F\left(u\right)={\left(2e\right)}^{\beta }\frac{\beta u}{{\left(2-u\right)}^{2+\beta }}\exp\left(\frac{-2\beta }{2-u}\right)$$

where *u* is *R/R_cr_*, *β* = 2 for a 2D system, and *β* = 3 for a 3D system [26]. When the initial GSD is a Hillert distribution, the numerical implementation of MFM maintains the initial Hillert distribution at all times, even as the number of grains decreases, as shown in Figure 3a–c. The effect of the initial GSD on the evolution of 3D grain-size distributions during normal grain growth is shown in Figure 3d. The log-normal and Hillert GSDs have been utilized as the initial conditions. Figure 3d shows a gradual change in the initial log-normal GSD profile towards a steady-state Hillert distribution. Once the Hillert distribution has been achieved, it remains unchanged for the rest of the simulation. Cruz-Fabiano et al. [69] demonstrated, using 2D grain-growth full-field simulations based on a finite-element formulation, that the classical Hillert model can accurately capture grain-growth kinetics for various initial GSDs. Kim et al. [70] demonstrated the evolution of GSD during the early stages of 3D grain growth, starting from different non-steady-state GSDs. They showed how the GSD profile at the initial stage shifts towards a steady state that almost perfectly matches the Hillert 3D distribution. Their simulation was limited to relatively short times because, at longer times, an insufficient number of grains remained in the system. It has been shown that, for long-time simulations, the accuracy of the simulation strongly relies on the number of grains used [51]. Therefore, the upsampling algorithm introduced in the present study resolves this issue by maintaining the number of necessary grains through the expansion of the equivalent simulation volume, allowing for a continuous simulation of grain growth almost indefinitely.

**Figure 4:**
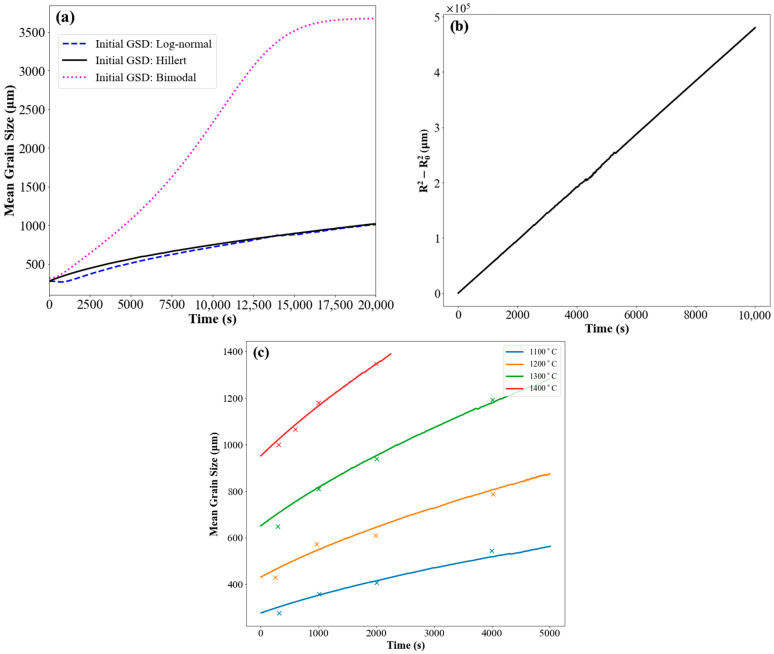
Computed temporal evolution of the number average mean grain size, using the initial grain-size distributions (GSDs) represented by the following: (**a**) log-normal (solid line), Hillert (dashed line), and bimodal (dotted) distribution. (**b**) Squared mean grain size changes linearly over time with the initial GSD like Hillert, showing the parabolic kinetics of grain growth. (**c**) The experimental austenite grain size (symbols) with a standard deviation of 11–23% of the measured mean grain-size values and the simulation results (solid lines) as a function of holding time at different temperatures of 1100 °C, 1200 °C, 1300 °C, and 1400 °C [66].

Figure 4a shows the effect of the initial GSD on grain-growth kinetics, using log-normal, Hillert, and bimodal distributions. In the case of an initial Hillert GSD, the growth kinetics were parabolic during the whole process. Parabolic kinetics are also observed for the simulation with a log-normal initial GSD. In this case, however, there is an initial transient region in which the kinetics are not parabolic. This was reported by other researchers and is associated with the evolution of the grain-size distribution from a non-steady-state distribution to the steady-state Hillert GSD [40]. The bimodal distribution resembled a Hillert distribution, but 5% of the total volume was assigned to grains with a mean radius size three times larger than the rest. The model was also capable of capturing abnormal grain growth when a small fraction of large grains ($R/R_{avg}$ > 3) were included within the initial GSD, as shown in Figure 4a. The rapid increase in the average size in this case was attributed to the consumption of the small grains by the large grains. This continued until the impingement of the large grains, which was followed by a process similar to normal grain growth which is referred to as post-abnormal grain growth [71,72]. Normally, numerical simulations would not be able to capture post-abnormal grain growth because a very small number of grains would remain within the simulation volume. The use of the upscaling algorithm, however, makes it possible to continue the simulation by ensuring that the required minimum number of grains is always included in the simulation. Figure 4b demonstrates the time evolution of the squared mean grain size. It was found by fitting to the curve the notion that the outputs obeyed the parabolic law in grain growth, ${\bar{R}}^{2}-{\bar{R}}_{0}^{2}=kt$, where *t* is time, *k* is the growth constant, and ${\bar{R}}_{0}$ is the average initial grain size [16]. At 1100 °C, the growth constant was calculated as *k* = $4.8\;\mu m^{2}s^{-1}$.

The average grain-size evolution of a micro-alloyed steel (~0.01 wt.% Nb,0.1 wt.% C, 0.97 wt.% Mn, 0.3 wt.% Si) as a function of holding time is shown in Figure 4c. The simulation results are plotted against the experimental data at temperatures in the range of 1100 °C to 1400 °C. The experimental data of the mean grain size reported by [66] were obtained using the linear intercept method after etching the samples to reveal the prior austenite grain boundaries. The three-dimensional grain diameter was estimated to be 1.61 times the diameter obtained by the linear intercept. The grain boundary mobility was determined by Equation (5), utilizing parameters from Nb micro-alloyed steel [66]. As it can be seen, the mean grain size and growth rate increased by increasing the temperature. The simulation outcomes align well with the experimental results derived from the grain-growth experiments.

One of the crucial aspects of modeling microstructure evolution is to maintain the efficiency of the model as well as the accuracy. It was discussed in the previous section that the number of grains in the system can dramatically change over time. In the case of grain growth, small grains will disappear from the ensemble, and, in the case of recrystallization, new grains will be added to the ensemble. This naturally leads to two undesired scenarios in which there are, respectively, too few or too many grains in the ensemble. In order to address this problem, a rescaling procedure was implemented as described above.

**Figure 5:**
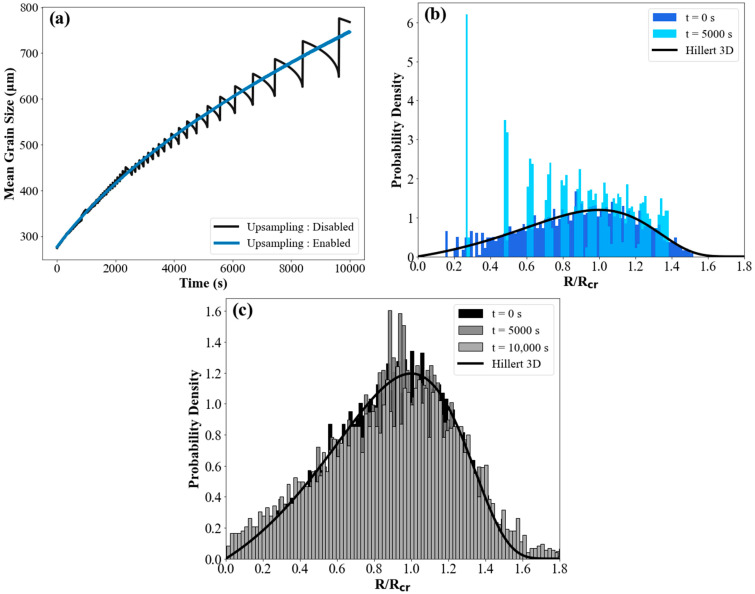
The effects of the dynamic adjustment of the number of grain bins on the following: (**a**) the mean grain size with the upsampling enabled versus disabled; (**b**) the GSD at different time points when the algorithm is disabled; and (**c**) the GSD at different time points when the algorithm is enabled.

A critical parameter of the simulation is the number of grain bins. This number should be sufficiently large to accurately capture the evolution of the grain size. In the case of grain growth, the number of grain bins decreases over time owing to the disappearance of the smaller grains. Figure 5a shows that numerical artefacts emerge at longer times because an insufficient number of grains remain in the system modeled in this study. In contrast, when the upsampling algorithm is implemented, the sample volume is continuously adjusted to ensure that a sufficient number of grains are present within this volume. As a result, accurate predictions of the average radius and size distribution are obtained even at very long simulation times.

Table 1 demonstrates that the simulation utilizing the upsampling algorithm exhibits the highest overall r-squared value and the lowest root mean square error (RMSE) when compared with the theoretical parabolic growth law. In contrast, when upsampling is not used, the r-squared and RMSE values deteriorate over time due to not having sufficient bins for an accurate simulation. To quantify this discussion, a moving standard deviation is computed over the entire simulation period to identify the time at which deviations from the theoretical values exceed the acceptable accuracy limit (i.e., moving standard deviation >5 μm). When the initial number of grains in the simulation is only 100, significant deviations from the theoretical predictions are observed after 1700 s. Increasing the initial number of grains to 1000 ensures a good fit for up to 6000 s. Beyond that time, the drop in the number of grains will again lead to large errors. When the upsampling algorithm is implemented, the accuracy of the simulation is maintained almost indefinitely.

Similarly, Figure 5b depicts the GSD under conditions where the algorithm is inactive, leading to the GSD deviating from a steady state as a consequence of a decrease in the grain count. This deviation is attributed to the small number of bins remaining. When the upscaling algorithm is used, new bins are added to ensure that an appropriate number of grains continue to be used in order to maintain the accuracy of the simulation. This ensures that a Hillert distribution is maintained throughout the process, as depicted in Figure 5c. In order to assess the impact of the upsampling algorithm on the computational efficiency of grain-growth simulations, 5000 starting bins were used to examine the extended duration of simulations compared to automatic simulations and identify when accuracy was compromised due to the inability to add bins during the growth experiments. Disabling the upsampling algorithms resulted in significantly increased simulation times and a lower accuracy. When upsampling is disabled, the declining number of grains during the simulation may lead to reduced running times, although the model accuracy is completely lost after 6000 s of simulation.

## 4. Conclusions

This study introduces a novel mean-field modeling approach for simulating the grain-growth process. The model was successfully tested against grain-growth theories that demonstrated its ability to accurately capture key aspects, including the parabolic growth pattern and the self-similarity in the grain-size distribution across different time points. The primary novelty of the model lies in its efficiency and ability to maintain accuracy even during prolonged simulations, achieved through the innovative algorithm for dynamically adjusting the number of grains. It was demonstrated that simulations using the upsampling algorithm achieved the lowest RMSE and highest r^2^ values for parabolic kinetics, showing the highest agreement with analytical models and allowing the simulation to run indefinitely. Ultimately, the validation of this model was conducted against experimental findings from grain-growth experiments involving micro-alloyed steels, demonstrating a very good agreement. This paper presents a robust algorithm capable of handling the various microstructures, including steady-state as well as and non-steady-state ones, which could emerge during thermomechanical processing conditions. The adaptive upsampling algorithm introduced in this study can be integrated into other models, enhancing efficiency as well as facilitating seamless incorporation with other components of microstructure evolution models. Future endeavors will focus on integrating this grain-growth model with other microstructural evolution phenomena such as dynamic and static recrystallization, with the aim of developing a comprehensive model for the evolution of grain size. Consequently, a through-process model of hot-rolling will be developed to incorporate microstructure evolution components. This model will track microstructure changes during thermomechanical processing and assist in optimizing and predicting product properties, ultimately aiding in the design of new products and the optimization of hot-rolling schedules.

## Figures and Tables

**Table 1:** Effect of ensemble size on the accuracy of the model output: t_max_ shows the longest simulation time during which accurate parabolic kinetics are observed. The root mean square error (RMSE) for each simulation is also included. The r^2^ values show the extent to which the calculated kinetics approach the parabolic growth law at 1100 °C and 0.01 wt.% Nb.

Final Ensemble Size	t_max_ (s)	r^2^	RMSE
Auto	NA	0.98	14.84
1000	6000	0.96	15.24
500	4000	0.96	16.38
100	1700	0.90	23.32

## Data Availability

Data are contained within the article.

## References

[1] Sellars C.M. (1990). Modelling microstructural development during hot rolling. Mater. Sci. Technol..

[2] Hodgson P.D. (1992). A mathematical model to predict the mechanical properties of hot rolled C-Mn and microalloyed steels. ISIJ Int..

[3] Hodgson P.D. (1993). Models of the recrystallization behaviour of C-Mn and Nb microalloyed steels during hot working processes. Met. Forum.

[4] Dunlop J.W.C., Bréchet Y.J.M., Legras L., Zurob H.S. (2007). Modelling isothermal and non-isothermal recrystallisation kinetics: Application to Zircaloy-4. J. Nucl. Mater..

[5] Rehman K., Zurob H.S. (2013). Novel approach to model static recrystallization of austenite during hot-rolling of Nb-Microalloyed steel: Effect of precipitates. Mater. Sci. Forum.

[6] Pietrzyk M. (2002). Through-process modelling of microstructure evolution in hot forming of steels. J. Mater. Process. Technol..

[7] Elder K.R., Katakowski M., Haataja M., Grant M. (2002). Modeling Elasticity in Crystal Growth. Phys. Rev. Lett..

[8] Militzer M., Hawbolt E., Meadowcroft T. (2000). Microstructural model for hot strip rolling of high-strength low-alloy steels. Metall. Mater. Trans. A.

[9] Zurob H.S., Hutchinson C.R., Brechet Y., Purdy G. (2002). Modeling recrystallization of microalloyed austenite: Effect of coupling recovery, precipitation and recrystallization. Acta Mater..

[10] Zurob H.S., Brechet Y., Purdy G. (2001). A model for the competition of precipitation and recrystallization in deformed austenite. Acta Mater..

[11] Hoffman A.K., Umretiya R.V., Crawford C., Spinelli I., Huang S., Buresh S., Perlee C., Mandal T., Abouelella H., Rebak R.B. (2023). The relationship between grain size distribution and ductile to brittle transition temperature in FeCrAl alloys. Mater. Lett..

[12] Atkinson H.V. (1988). Overview no. 65: Theories of normal grain growth in pure single phase systems. Acta Metall..

[13] Shirdel M., Mirzadeh H., Habibi Parsa M. (2014). Microstructural evolution during normal/abnormal grain growth in austenitic stainless steel. Metall. Mater. Trans. A.

[14] Benson W., Wert J. (1998). The effect of initial grain size distribution on abnormal grain growth in single-phase materials. Acta Mater..

[15] Krill III C.E., Holm E.A., Dake J.M., Cohn R., Holíková K., Andorfer F. (2023). Extreme Abnormal Grain Growth: Connecting Mechanisms to Microstructural Outcomes. Annu. Rev. Mater. Res..

[16] Burke J.E., Turnbull D. (1952). Recrystallization and grain growth. Prog. Met. Phys..

[17] Smith C. (1952). Grain Shapes and Other Metallurgical Applications of Topology Metal Interfaces.

[18] Feltham P. (1957). Grain growth in metals. Acta Metall..

[19] Louat N.P. (1974). On the theory of normal grain growth. Acta Metall..

[20] Enomoto M., Hayashi K. (2023). Estimation of austenite grain boundary mobility in low-carbon steel by grain growth. J. Mater. Sci..

[21] Abbruzzese G., Lücke K. (1986). A theory of texture controlled grain growth—I. Derivation and general discussion of the model. Acta Metall..

[22] Eichelkraut H., Abbruzzese G., Lücke K. (1988). A theory of texture controlled grain growth—II. Numerical and analytical treatment of grain growth in the presence of two texture components. Acta Metall..

[23] Wu K., Jeppsson J., Mason P. (2022). Mean Field Modeling of Grain Growth and Zener Pinning. J. Phase Equilibria Diffus..

[24] Maire L., Scholtes B., Moussa C., Bozzolo N., Pino Muñoz D., Bernacki M. (2016). Improvement of 3D mean field models for capillarity-driven grain growth based on full field simulations. J. Mater. Sci..

[25] Roth M., Flipon B., Bozzolo N., Bernacki M. (2023). Comparison of Grain-Growth Mean-Field Models Regarding Predicted Grain Size Distributions. Materials.

[26] Hillert M. (1965). On the theory of normal and abnormal grain growth. Acta Metall..

[27] Abbruzzese G., Heckelmann I., Lücke K. (1992). Statistical theory of two-dimensional grain growth—I. The topological foundation. Acta Metall. Mater..

[28] Maire L., Fausty J., Bernacki M., Bozzolo N., De Micheli P., Moussa C. (2018). A new topological approach for the mean field modeling of dynamic recrystallization. Mater. Des..

[29] Andersson J.-O., Helander T., Höglund L., Shi P., Sundman B. (2002). Thermo-Calc & DICTRA, computational tools for materials science. Calphad.

[30] Upmanyu M., Hassold G.N., Kazaryan A., Holm E.A., Wang Y., Patton B., Srolovitz D.J. (2002). Boundary mobility and energy anisotropy effects on microstructural evolution during grain growth. Interface Sci..

[31] Bulatov V.V., Reed B.W., Kumar M. (2014). Grain boundary energy function for fcc metals. Acta Mater..

[32] Darvishi Kamachali R., Steinbach I. (2012). 3-D phase-field simulation of grain growth: Topological analysis versus mean-field approximations. Acta Mater..

[33] Rios P.R. (1999). Comparison between a grain size distribution obtained by a Monte Carlo Potts model and by an analytical mean field model. Scr. Mater..

[34] Blikstein P., Tschiptschin A.P. (1999). Monte Carlo simulation of grain growth. Mater. Res..

[35] Xiaoyan S., Guoquan L., Nanju G. (2000). Re-analysis on grain size distribution during normal grain growth based on Monte Carlo simulation. Scr. Mater..

[36] Zöllner D., Streitenberger P. (2006). Three-dimensional normal grain growth: Monte Carlo Potts model simulation and analytical mean field theory. Scr. Mater..

[37] Ivasishin O.M., Shevchenko S.V., Vasiliev N.L., Semiatin S.L. (2003). 3D Monte-Carlo simulation of texture-controlled grain growth. Acta Mater..

[38] Brakke K.A. (1992). The surface evolver. Exp. Math..

[39] Marthinsen K., Hunderi O., Ryum N. (1996). The influence of spatial grain size correlation and topology on normal grain growth in two dimensions. Acta Mater..

[40] Wakai F., Enomoto N., Ogawa H. (2000). Three-dimensional microstructural evolution in ideal grain growth—General statistics. Acta Mater..

[41] Weygand D., Bréchet Y., Lépinoux J., Gust W. (1999). Three-dimensional grain growth: A vertex dynamics simulation. Philos. Mag. B.

[42] Fuchizaki K., Kusaba T., Kawasaki K. (1995). Computer modelling of three-dimensional cellular pattern growth. Philos. Mag. B.

[43] Marsh S., Masumura R., Pande C. (1995). A curvature-driven vertex model for two-dimensional grain growth. Philos. Mag. Lett..

[44] Fullman R. (1952). Boundary migration during grain growth. Met. Interfaces.

[45] Bronsard L., Wetton B.T. (1995). A numerical method for tracking curve networks moving with curvature motion. J. Comput. Phys..

[46] Frost H.J., Thompson C.V. (1996). Computer simulation of grain growth. Curr. Opin. Solid. State Mater. Sci..

[47] Fayad W., Thompson C., Frost H. (1999). Steady-state grain-size distributions resulting from grain growth in two dimensions. Scr. Mater..

[48] Krill Iii C.E., Chen L.Q. (2002). Computer simulation of 3-D grain growth using a phase-field model. Acta Mater..

[49] Yadav V., Vanherpe L., Moelans N. (2016). Effect of volume fractions on microstructure evolution in isotropic volume-conserved two-phase alloys: A phase-field study. Comput. Mater. Sci..

[50] Yadav V., Moelans N. (2018). Investigation on the existence of a ‘Hillert regime’in normal grain growth. Scr. Mater..

[51] Gao J., Wei M., Zhang L., Du Y., Liu Z., Huang B. (2018). Effect of Different Initial Structures on the Simulation of Microstructure Evolution During Normal Grain Growth via Phase-Field Modeling. Metall. Mater. Trans. A.

[52] Szeliga D., Bzowski K., Rauch Ł., Kuziak R., Pietrzyk M. (2020). Mean field and full field modelling of microstructure evolution and phase transformations during hot forming and cooling of low carbon steels. Comp. Methods Mater. Sci..

[53] Després A., Greenwood M., Sinclair C. (2020). A mean-field model of static recrystallization considering orientation spreads and their time-evolution. Acta Mater..

[54] Cram D., Zurob H.S., Brechet Y., Hutchinson C. (2009). Modelling discontinuous dynamic recrystallization using a physically based model for nucleation. Acta Mater..

[55] Piot D., Smagghe G., Jonas J.J., Desrayaud C., Montheillet F., Perrin G., Montouchet A., Kermouche G. (2018). A semitopological mean-field model of discontinuous dynamic recrystallization: Toward a correct and rapid prediction of grain-size distribution. J. Mater. Sci..

[56] Anderson M., Srolovitz D., Grest G., Sahni P. (1984). Computer simulation of grain growth—I. Kinetics. Acta Metall..

[57] Ponge D., Gottstein G. (1998). Necklace formation during dynamic recrystallization: Mechanisms and impact on flow behavior. Acta Mater..

[58] Montheillet F., Lurdos O., Damamme G. (2009). A grain scale approach for modeling steady-state discontinuous dynamic recrystallization. Acta Mater..

[59] Darvishi Kamachali R., Abbondandolo A., Siburg K.F., Steinbach I. (2015). Geometrical grounds of mean field solutions for normal grain growth. Acta Mater..

[60] Rios P.R., Dalpian T.G., Brandão V.S., Castro J.A., Oliveira A.C.L. (2006). Comparison of analytical grain size distributions with three-dimensional computer simulations and experimental data. Scr. Mater..

[61] Dumitrescu D., Boiangiu C.-A. (2019). A Study of Image Upsampling and Downsampling Filters. Computers.

[62] Unser M., Aldroubi A., Eden M. (1993). B-spline signal processing. II. Efficiency design and applications. IEEE Trans. Signal Process..

[63] Fattal R. (2007). Image upsampling via imposed edge statistics. ACM SIGGRAPH 2007 Papers.

[64] Monno Y., Tanaka M., Okutomi M. Multispectral demosaicking using adaptive kernel upsampling. Proceedings of the 2011 18th IEEE International Conference on Image Processing.

[65] Li Y., Liu D., Li H., Li L., Wu F., Zhang H., Yang H. (2017). Convolutional neural network-based block up-sampling for intra frame coding. IEEE Trans. Circuits Syst. Video Technol..

[66] Furumai K., Wang X., Zurob H., Phillion A. (2019). Evaluating the Effect of the Competition between NbC Precipitation and Grain Size Evolution on the Hot Ductility of Nb Containing Steels. ISIJ Int..

[67] Zhou T., O’malley R.J., Zurob H.S. (2010). Study of grain-growth kinetics in delta-ferrite and austenite with application to thin-slab cast direct-rolling microalloyed steels. Metall. Mater. Trans. A.

[68] Cahn J.W. (1962). The impurity-drag effect in grain boundary motion. Acta Metall..

[69] Cruz-Fabiano A.L., Logé R., Bernacki M. (2014). Assessment of simplified 2D grain growth models from numerical experiments based on a level set framework. Comput. Mater. Sci..

[70] Kim S.G., Kim D.I., Kim W.T., Park Y.B. (2006). Computer simulations of two-dimensional and three-dimensional ideal grain growth. Phys. Rev. E.

[71] Humphreys F.J., Hatherly M. (2012). Recrystallization and Related Annealing Phenomena.

[72] Zhou T.H., Zurob H.S. (2011). Abnormal and post-abnormal austenite grain growth kinetics in Nb–Ti microalloyed steels. Can. Metall. Q..

